# Expression of Animal Anti-Apoptotic Gene Ced-9 Enhances Tolerance during *Glycine max* L.–*Bradyrhizobium japonicum* Interaction under Saline Stress but Reduces Nodule Formation

**DOI:** 10.1371/journal.pone.0101747

**Published:** 2014-07-22

**Authors:** Germán Robert, Nacira Muñoz, Mariana Melchiorre, Federico Sánchez, Ramiro Lascano

**Affiliations:** 1 Instituto de Fisiología y Recursos Genéticos Vegetales, Centro de Investigaciones Agropecuarias-INTA, Córdoba, Argentina; 2 Cátedra de Fisiología Vegetal, Facultad de Ciencias Exactas Físicas y Naturales, Universidad Nacional de Córdoba, Córdoba, Argentina; 3 Departamento de Biología Molecular de Plantas, Instituto de Biotecnología, Universidad Nacional Autónoma de México, Cuernavaca, Morelos, México; Institute of Genetics and Developmental Biology, Chinese Academy of Sciences, China

## Abstract

The mechanisms by which the expression of animal cell death suppressors in economically important plants conferred enhanced stress tolerance are not fully understood. In the present work, the effect of expression of animal antiapoptotic gene Ced-9 in soybean hairy roots was evaluated under root hairs and hairy roots death-inducing stress conditions given by i) *Bradyrhizobium japonicum* inoculation in presence of 50 mM NaCl, and ii) severe salt stress (150 mM NaCl), for 30 min and 3 h, respectively. We have determined that root hairs death induced by inoculation in presence of 50 mM NaCl showed characteristics of ordered process, with increased ROS generation, MDA and ATP levels, whereas the cell death induced by 150 mM NaCl treatment showed non-ordered or necrotic-like characteristics. The expression of Ced-9 inhibited or at least delayed root hairs death under these treatments. Hairy roots expressing Ced-9 had better homeostasis maintenance, preventing potassium release; increasing the ATP levels and controlling the oxidative damage avoiding the increase of reactive oxygen species production. Even when our results demonstrate a positive effect of animal cell death suppressors in plant cell ionic and redox homeostasis under cell death-inducing conditions, its expression, contrary to expectations, drastically inhibited nodule formation even under control conditions.

## Introduction

Programmed cell death (PCD) is a genetically regulated process of cellular suicide and is well known to play a fundamental role in a wide variety of developmental and physiological functions in animals, plants, and fungi [1]–[3]. A key feature of PCD is the requirement of energy to the control and execution of the death in an orderly manner [4], [5]. In plants, PCD is essential for cell homeostasis and specialization, playing an important role in plant development and adaptive responses to different stress conditions such as salinity, cold stress, hypoxia and pathogen attack [6]–[8].

In metazoans, from humans to *Caenorhabditis elegans*, the central regulators of PCD are well characterized and conserved involving pro- and anti-apoptotic protein such as APAF-1/CED-4 and BCL-2/CED-9, and executing protein family caspasas/CED-3 [9], [10]. Interestingly, although these regulators are absent in the genomes of plants and yeast, the effects of animal pro- and anti-apoptotic proteins has been studied in transgenic plants [11]–[18]. According to the localization of these heterologous proteins in plant cells, it is proposed that cell death suppressors contribute to maintain the organelles homeostasis preventing the generation/release of death signals, similar to what occurs in animals [11], [13]. However, there are limited data regarding the mechanisms through which the animal cell death suppressors modulate the plant physiology.

Remarkably, the expression of PCD suppressors in plants result in agronomical beneficial features such as improved tolerance to a variety of biotic and abiotic stresses [11]–[16]. Increased the biological nitrogen fixation in legumes is a main objective for the agriculture, and different strategies had been explored towards this objective. During the natural or stress induced senescence, which involve cell death processes, the biological nitrogen fixation metabolism is impaired, affecting both quality and quantity of legume yields [19], [20]. Therefore, the development of strategies to increase the tolerance to a variety of stresses is highly relevant. To the best of our knowledge, the effect of animal PCD suppressor has not been tested in legumes.

Soybean (*Glycine max* L.) culture is strongly affected by drought and salinity [21], [22]. Likewise, the soybean-rhizobia symbiotic interaction process is also severely affected by stress conditions [19], [20]. Our group has reported that salt stress, but not osmotic stress, negatively affect the early stages of the *Glycine max* L.-*Bradyrhizobium japonicum* interaction such as root hairs deformations and viability [23], and how these short-term treatments affect nodule formation [24]. In this context, two root hairs death-inducing conditions were identified: sub lethal salt stress treatments combined with *B. japonicum* inoculation (inoculated 50 mM NaCl) and severe salt stress (150 mM NaCl). Interestingly, in root hairs under sub lethal salt stress conditions (50 mM NaCl), the symbiotic interaction with *B. japonicum* induced a sustained increase of intracellular reactive oxygen species (ROS) levels in a similar pattern to that observed in response to pathogenic elicitors [23], [25]. In contrast, under 150 mM NaCl, the intracellular ROS production decreased from the beginning of treatment independently of the presence of the symbiont [23].

The aim of the present work was to evaluate if the expression of Ced-9 from *Caenorhabditis elegans* could improve the stress tolerance of legume-rizobia symbiotic interaction and the biological nitrogen fixation process. Transgenic soybean-hairy roots expressing Ced-9, obtained with Agrobacterium rhizogenes [26], [27], were subjected to the above described cell death-inducing conditions, in order to evaluate root cell viability, redox and ionic parameters associated and nodule development.

## Results

### Root hairs death-inducing stress conditions

**Figure 1:**
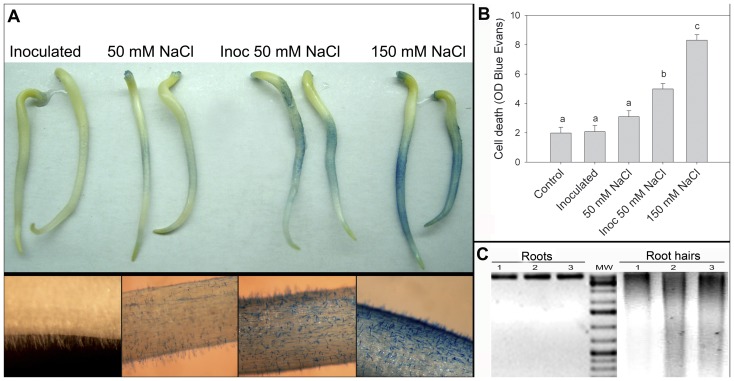
Root hairs death inducing stress conditions. Two-day soybean seedlings were subjected 30 min to control, inoculated with *B. japonicum* (inoculated), inoculated with *B. japonicum* in presence of 50 mM NaCl (inoc 50 mM NaCl), and 150 mM NaCl conditions. A) Evans blue staining of roots showing loss of membrane integrity (upper image) and detail of root hairs staining (image below). B) Evans Blue stain intensity was measured by the image analyzer program Optimas 6.1. C) DNA degradation in a representative sample pool of root and root hairs. 1: Control, 2: inoculated 50 mM NaCl, 3: 150 mM NaCl. 2 µg of DNA were loaded on a 2% TAE agarose gel and stained with ethidium bromide. Data are means ± SE of four independent experiments (two roots per experiment). Different letters indicate significant differences between treatments (p<0.05, DGC test).

Two-day soybean seedlings were subjected 30 min under previously reported root hairs death-inducing conditions. Moderate salt stress treatments (50 mM NaCl) combined with *B. japonicum* (inoculated 50 mM NaCl) and severe salt stress (150 mM NaCl) were the cell death conditions for root hairs [23]. However, under these root hairs death-inducing conditions the roots were kept alive. These results were observed by Evans Blue staining and DNA degradation analysis (Fig. 1A, 1B and 1C). Root hairs DNA degradation was observed while roots maintained the chromatin integrity (Fig. 1C).

**Figure 2:**
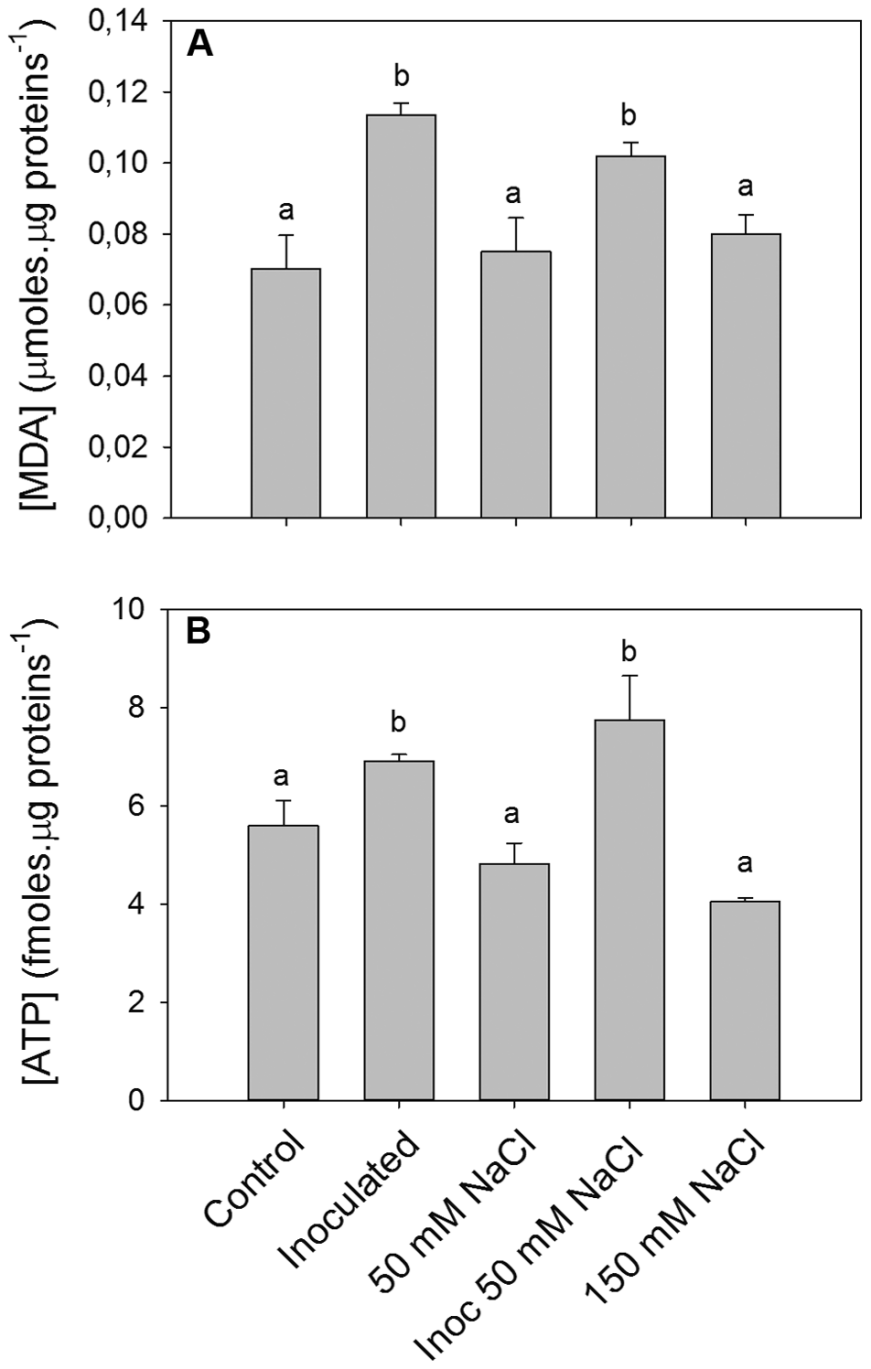
Evaluation of malondialdehyde (MDA) and Adenosine-5′-triphosphate (ATP) levels in root hairs. Two-day soybean seedlings were subjected 30 min to control, inoculated with *B. japonicum* (inoculated), 50 mM NaCl, inoculated with *B. japonicum* in presence of 50 mM NaCl (inoc 50 mM NaCl), and 150 mM NaCl conditions. Then, root hairs were extracted from roots and MDA and ATP content were evaluated. Data are means ± SE of four (MDA) and three (ATP) independent experiments (root hairs from 200 roots per experiment). Different letters indicate significant differences between treatments (p<0.05, DGC test).

Malondialdehyde content (MDA), which is an intermediary metabolite of lipid peroxidation used as oxidative stress marker, was measured in root hairs. The MDA level increased in inoculated and inoculated 50 mM NaCl treatments, whereas non significant differences were observed in salt stress alone (50 mM NaCl and 150 mM NaCl) respect to control (Fig. 2A). Likewise, with the purpose to discriminate ordered or non-ordered death processes, the levels of adenosine-5′-triphosphate (ATP) were quantified [4], [28] in root hairs subjected to root hairs death-inducing conditions. No significant changes were observed at 50 mM NaCl (Fig. 2B). Under inoculated 50 mM NaCl treatment, root hairs had increased ATP levels as well as under inoculated control treatment (Fig. 2B). Conversely, under 150 mM NaCl treatment, root hairs showed a slight, but not significant decrease in ATP levels respect to the control (Fig. 2B).

### CED-9 expression ameliorates root hairs death-inducing conditions effects

In order to evaluate the effect of the animal cell death suppressor, wild type and CED-9 transgenic hairy roots were obtained by infection with *A. rhizogenes* K599 strain. It should be pointed out that the differentiation and development of hairy roots were conducted without antibiotic selection, thus the resulting K599-CED9 composite plants contained both transgenic and wild type hairy roots (Fig. S1A). The expression level of the transgene in hairy roots was tested by qPCR using Ced-9 specific-derived primers (Fig. S1B) and the identity of qPCR product was verified by nucleotide sequencing. The K599-CED9 hairy roots were shorter than wild type hairy roots obtained by infection with untransformed *A. rhizogenes* (K599-empty) (Fig. S2A).

**Figure 3:**
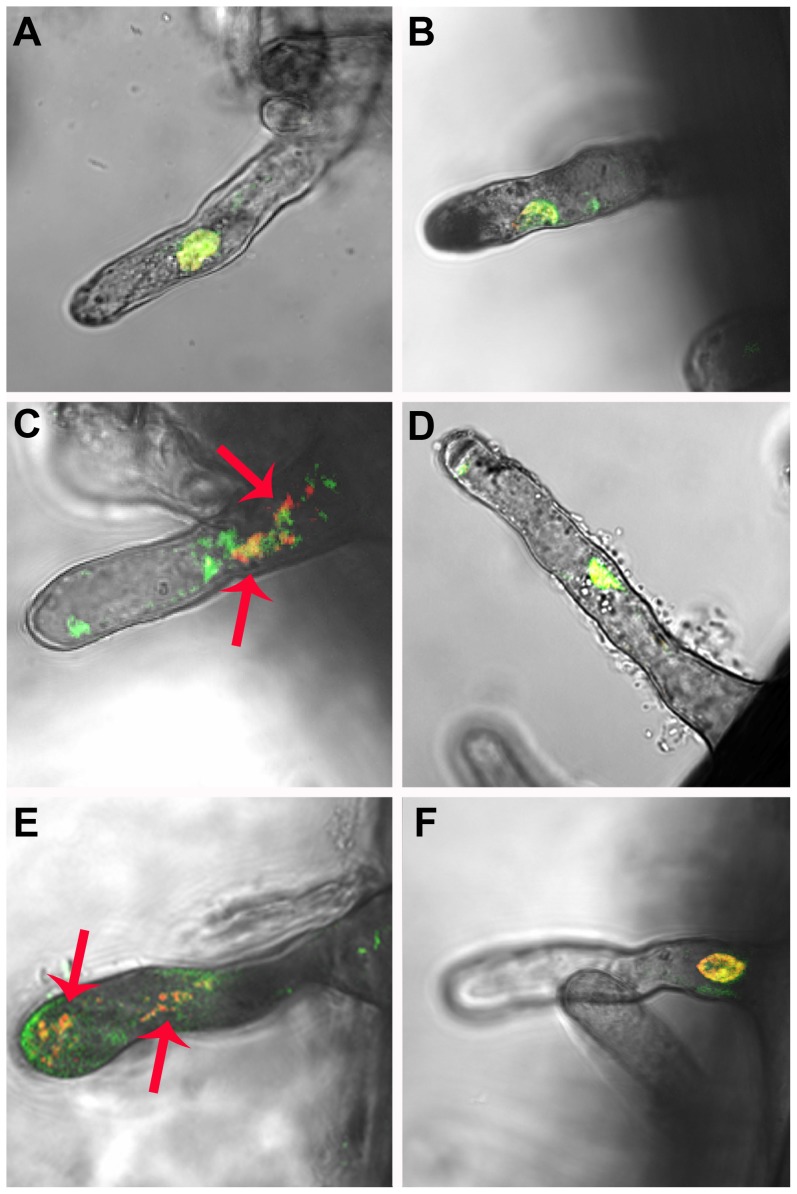
Ced-9 expression ameliorates root hairs death-inducing conditions effects. K599-empty (A, C, E) and K599-CED9 (B, D, F) hairy roots were subjected 30 min under control (A and B), inoculated with *B. japonicum* in presence of 50 mM NaCl (inoc 50 mM NaCl) (C and D), and 150 mM NaCl (E and F). Nuclear morphology of root hairs was evaluated. Arrows indicates nuclear fragmentation. Images were taken with a Zeiss confocal microscope. The excitation was performed simultaneously at 488 nm and emission filter BP 500–530 IR and BP 565–615 IR for AO and EtBr, respectively (image overlay).

Roots hairs nuclear morphology was evaluated in K599-empty and K599-CED9 hairy roots incubated 30 min under control, or root hair death-inducing conditions (Fig. 3; Fig. S3). The nuclear morphology of root hairs was evaluated by acridine orange and ethidium bromide (AO/EB) staining and observed with confocal microscopy. Acridine orange is a dual-fluorescence dye that interacts with DNA and RNA, and it also serves as a pH indicator. Ethidium bromide binds to DNA by intercalating between the bases, but it is membrane impermeant so generally excluded from viable cells [28]. Hence, roots were incubated 30 min with AO/EtBr to allow entry of the probes. The nuclei of root hairs in the control treatments exhibited an orthodox conformation, and similar size and shape between K599-empty and K599-CED9 hairy roots (Fig. S3). Under root hair death-inducing conditions, root hairs of K599-empty hairy roots showed significantly higher nuclear fragmentation than root hairs of K599-CED9 hairy roots (Fig. 3). Furthermore, an increase in AO staining was observed in K599-empty hairy roots particularly under 150 mM NaCl conditions (Fig. 3).

### CED-9 effects on root ion and redox homeostasis under hairy roots death-inducing conditions

Whole hairy roots were used to perform the biochemical determinations due to the low yield of root hairs in hairy roots. However, as previously observed, root hair death-inducing conditions did not induce roots death. Then, treatment time was adjusted to 3 h, when positive Evans Blue staining (Fig. S2A), but not DNA degradation (Fig. S2B) could be observed. This result indicates an early stage of root cell death. It also was noted that K599-CED9 hairy roots showed more membrane selectivity than K599-empty hairy roots (Fig. S2A).

**Figure 4:**
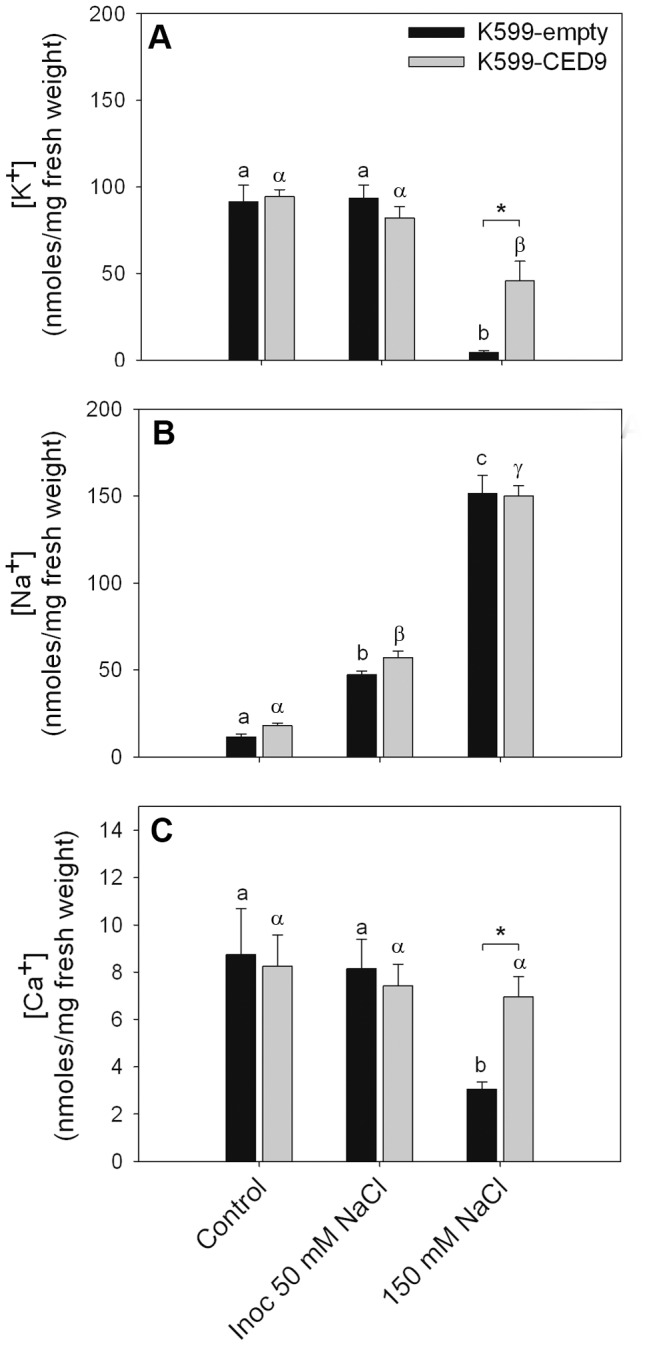
Ced-9 affects ion relationship during hairy roots death-inducing stress conditions. K599-empty (dark bars) and K599-CED9 (grey bars) hairy roots were subjected 3 h to control, inoculated with *B. japonicum* in presence of 50 mM NaCl (inoc 50 mM NaCl), and 150 mM NaCl conditions, and then potassium, sodium and calcium ions were quantified by high pressure liquid chromatography. Data are means ± SE of five independent hairy roots. Different Latin and Greek letters indicate significant differences between treatments in K599-empty and K599-CED9 hairy roots, respectively (p<0.05, DGC test). Asterisks indicate significant differences between hairy roots genotypes (p<0.05, DGC test).

K599-empty hairy roots showed a dramatic decrease in potassium levels under 150 mM NaCl treatment, whereas in K599-CED9 hairy roots this decrease was much less pronounced. Nevertheless, no significant differences were observed in potassium content between control and inoculated 50 mM NaCl treatments in any transgenic or wild type genotype (Fig. 4A). However, sodium content in hairy roots increased in a dose dependent manner in both K599-empty and K599-CED9 hairy roots (Fig. 4B). Moreover, no significant differences in the concentration of sodium were observed between K599-empty and K599-CED9 in any of the treatments performed (Fig. 4B). Likewise, calcium concentration decreased markedly in K599-empty hairy roots only under 150 mM NaCl treatment (Fig. 4C), whereas the calcium content in K599-CED-9 hairy roots did not change in stress treatment respect to control (Fig. 4C).

**Figure 5:**
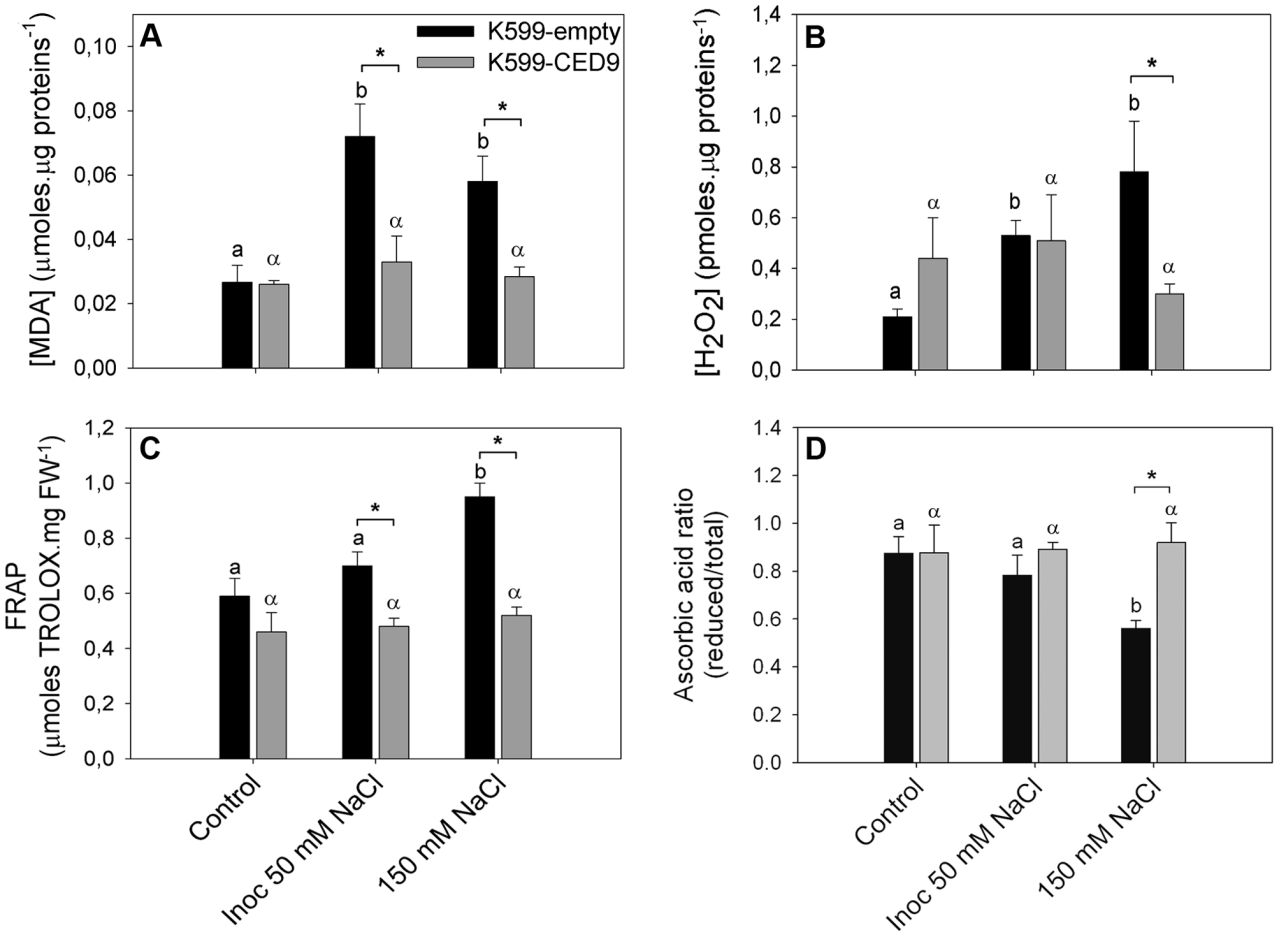
Ced-9 expression prevents ROS generation under hairy roots death-inducing stress conditions. K599-empty (dark bars) and K599-CED9 (grey bars) hairy roots were subjected 3 h under control, inoculated with *B. japonicum* in presence of 50 mM NaCl (inoc 50 mM NaCl), and 150 mM NaCl conditions, and redox parameters were evaluated. A) MDA content, B) H_2_O_2_ content, C) FRAP (Ferric Reducing Ability of Plasma) assay, D) Reduced/total ascorbic acid ratio. Data are means ± SE from five independent hairy roots. Different Latin and Greek letters indicate significant differences between treatments in K599-empty and K599-CED9 hairy roots, respectively (p<0.05, DGC test). Asterisks indicate significant differences between hairy roots genotypes (p<0.05, DGC test).

**Figure 6:**
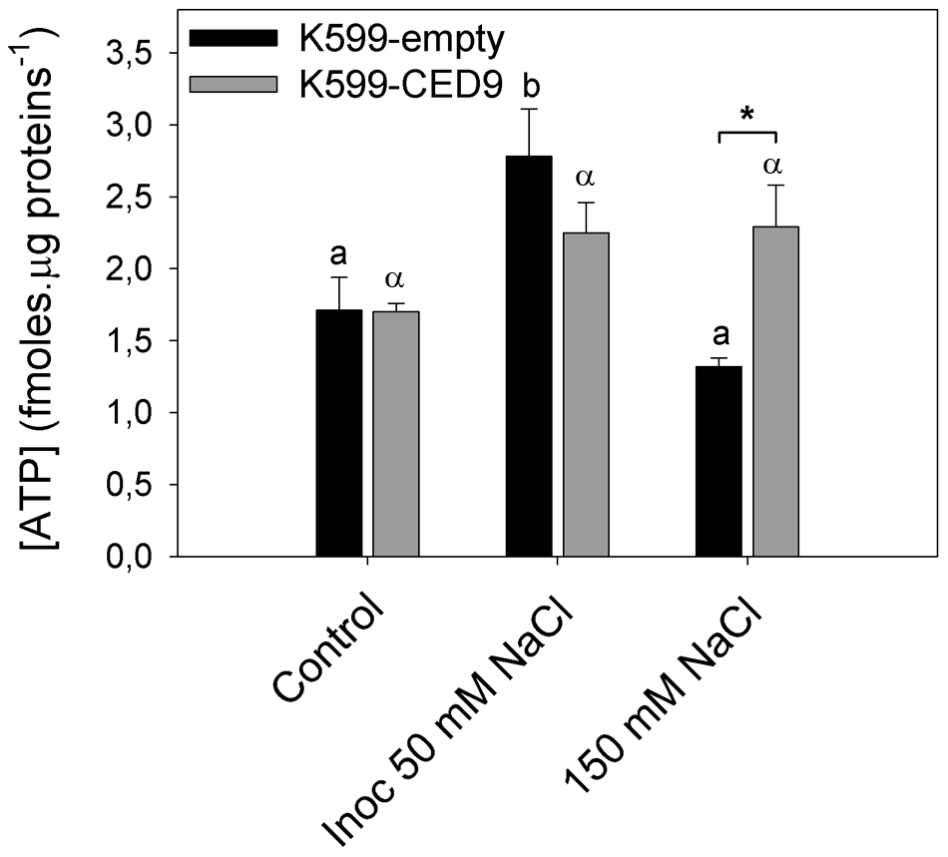
Effects of Ced-9 expression on adenosine-5′-triphosphate (ATP) levels in control and stressed hairy roots. K599-empty (dark bars) and K599-CED9 (grey bars) hairy roots were subjected 3 h to control, inoculated with *B. japonicum* in presence of 50 mM NaCl (inoc 50 mM NaCl), and 150 mM NaCl conditions and ATP content was evaluated. Data are means ± SE of four independent hairy roots. Different Latin and Greek letters indicate significant differences between treatments in K599-empty and K599-CED9 hairy roots, respectively (p<0.05, DGC test). Asterisks indicate significant differences between hairy roots genotypes (p<0.05, DGC test).

In order to characterize changes in the redox state during hairy roots death-inducing conditions, the levels of MDA, hydrogen peroxide (H_2_O_2_), antioxidant capacity, ascorbic acid ratio (reduced form/total) and ATP in K599-empty and K599-CED-9 hairy root were quantified (Fig. 5 and Fig. 6). MDA content increased in K599-empty hairy roots under stress conditions (Fig. 5A). Interestingly, K599-CED9 hairy roots did not show significant differences under any stress treatments regarding control (Fig. 5A). In accordance, H_2_O_2_ levels increased in K599-empty hairy roots under inoculated 50 mM NaCl and 150 mM NaCl treatments, whereas K599-CED9 hairy roots did not show significant differences in H_2_O_2_ content between control and stress treatments (Fig. 5B). Moreover, differences in H_2_O_2_ content between K599-empty and K599-CED9 were only observed under 150 mM NaCl treatment (Fig 5B). The Ferric Reducing Ability of Plasma (FRAP) assay showed raises in antioxidant capacity in K599-empty hairy roots under 150 mM NaCl (Fig. 5C), although the ascorbic acid ratio was reduced (Fig. 5D). Under roots death-inducing conditions, K599-CED9 hairy roots did not show differences in both antioxidant capacity and ascorbic acid ratio respect to control (Fig. 5C and 5D).

ATP levels increased in K599-empty hairy roots inoculated in presence of 50 mM NaCl treatment while no significant difference were observed under 150 mM NaCl (Fig. 6), similarly to the results observed in root hairs (Fig. 2). In contrast, K599-CED9 hairy roots had increased ATP content under all stress conditions, including 150 mM NaCl treatment, showing significant differences respect to K599-empty hairy roots (Fig. 6).

### Ced-9 expression inhibit nodule formation in hairy roots

**Figure 7:**
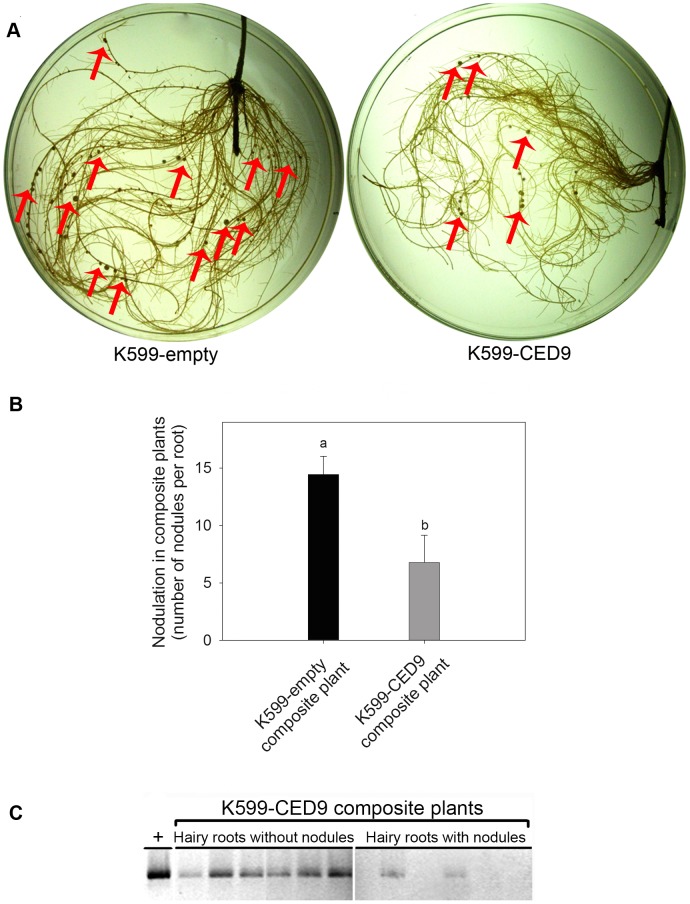
Ced-9 expression inhibits nodule formation in hairy roots. K599-empty and K599-CED9 composite plants were inoculated with *B. japonicum* USDA138 and after 14 days the nodulation was evaluated. A) Image of nodules in K599-empty and K599-CED9 hairy roots. B) Number of nodules in K599-empty (black bar) and K599-CED9 (grey bar) composite plants. Total nodules were counted and divided by the total number of hairy roots, including hairy roots without nodules (nodule number per hairy root). C) Nodulated and non-nodulated hairy roots of K599-CED9 composite plants were separated and Ced-9 transgenic hairy roots were identified by Ced-9 PCR (+ indicates Ced-9 positive control). Data are means ± SE of four composite plants. Different letters indicate significant differences between composite plant genotypes (p<0.05, DGC test).

Strikingly, K599-CED9 composite plants showed a reduction of 60% in the number of nodules compared to K599-empty composite plants under control conditions (Fig. 7A and 7B). As it was previously mentioned, each soybean K599-CED9 composited plants developed both transgenic K599-CED9 and non-transgenic hairy roots (Fig. S1). In the nodulation assay, K599-CED9 composite plants had both hairy roots with and without nodules. Hairy roots from K599-CED9 composite plants were separated according nodulated and non nodulated and the presence of Ced-9 transgene was examined by PCR. This experiment clearly showed that in K599-CED9 hairy roots nodulation was dramatically inhibited, while in the same composite plant, those non-transgenic hairy roots were nodulated (Fig. 7C).

## Discussion

Soybean-rhizobia symbiotic interaction is severely affected by salt stress, showing a reduction on number and weight of nodules in plants salinized with 26 mM NaCl [19], [20]. Our group had studied the effects of salt stress conditions on early events of *Glycine max* L.-*B. japonicum* symbiotic interaction, where undescribed root hairs death-inducing conditions were identified: sub lethal salt stress treatments combined with *B. japonicum* (inoculated 50 mM NaCl) and severe salt stress (150 mM NaCl) (Figure 1). During the early events of symbiotic interaction, a fast and transient increase of intracellular ROS generation take place in root hairs [23], [25], whereas a sustained ROS production was reported when the symbiotic interaction occurred under 50 mM NaCl [23]. A similar root hair ROS kinetic was observed in response to pathogenic elicitors [25]. In contrast, under 150 mM NaCl conditions, intracellular ROS production diminished from the beginning of treatment [23]. Hence, the initial hypothesis was that the expression of anti-apoptotic proteins from animals, which have no homologues identified in plants, modulates redox homeostasis and delays senescence and death processes of the plant-symbiont system in legumes under stress conditions.

First at all, in this work we characterized these two root hairs death-inducing conditions. There are two main ways to execute cell death: ordered (programmed-like) and non-ordered (necrosis). The ATP level is a fine parameter to distinguish ordered from non-ordered cell death types in mammalian cells [4], [5]. During apoptosis, ATP has to be maintained high to allow the formation of the apoptosome [29], [30], while ATP depletion has been observed during necrosis [31]–[33]. However, this observation has not been clearly observed in plants [34]–[37]. Casolo et al [38] found that in soybean cell cultures, low H_2_O_2_ concentration induces PCD, which is accompanied by a slight decrease in ATP. In addition, ATP depletion after PCD induction in *A. thaliana* [39] and tobacco BY-2 cells [40] has also been reported. It has been reported that environmental stimuli can produce different types of cell death depending on the stimulus intensity and the ATP availability within the cell [41]. Here, we have determined that root hairs death induced by inoculation in presence of 50 mM NaCl showed characteristics of ordered-process, with increased ROS generation, MDA and ATP levels, whereas the cell death induced by 150 mM NaCl treatment showed non-ordered or necrotic-like characteristics, like decreases in ROS production and ATP levels (Figure 2). Furthermore, the differences observed in Evans Blue staining between these death-inducing treatments (Figure 1A) also indicates the differences in the stress intensity which would lead to the execution of ordered-death or necrosis-like processes. Moreover, the increased MDA and ATP levels observed in control inoculated root hairs (Figure 2) would be due to the increased metabolic activity during early responses of the symbiotic interaction [25], [42], [43].

The expression of cell death suppressor Ced-9 from *C. elegans* inhibited or at least delayed cell death under root hairs death-inducing conditions (Figure 3). Furthermore, an increase in AO staining was observed especially under 150 mM NaCl conditions, which would indicate cellular acidification. Interestingly, the expression of Ced-9 affected both ordered and necrotic-like death events in root hairs (Figure 3), and it has also been documented in animal systems [44]–[46], suggesting similar functionality level between the components of the mechanisms of cell death in plants and animals. However, few works have evaluated homeostatic and physiological parameters in transgenic plants in order to understand the effects of the expression of Ced9. Shabala and coworkers [16] have demonstrated that the expression of Ced-9 delays the onset of leaf senescence symptoms under salt and oxidative stress conditions altering the flow patterns of K^+^ and H^+^ across the plasma membrane. Consistent with Shabala's results [16], K599-CED9 hairy roots showed altered potassium content respect K599-empty hairy roots only under 150 mM NaCl condition, whereas non-significant differences were observed under inoculated 50 mM NaCl between transgenic and wild type hairy roots. This result indicates different causes of death and therefore, different mechanisms of action of CED-9 under these stress conditions (Figure 4B). Moreover, CED-9 effects on K^+^-efflux may be due to sustained levels of Ca^2+^ which subsequently may affect the opening and closing balance of non-selective cation channels (NSCC) [47], [48] (Figure 4C), but Ca^2+^ subcellular localization approaches are required to verify this hypothesis. However, Ced9-expression had no effect on sodium influx which increased in dose dependent manner, similarly to that observed in K599-empty hairy roots (Figure 4B).

It has been shown that under saline stress ROS generation are induced [49]–[51] leading to oxidative damage [52], [53]. In this regard, it has been suggested that anti-apoptotic genes from animals would suppress ROS generation or promote its removal in plants [7], [14]. However, to best of our knowledge, there are no redox studies to support this hypothesis since these conclusions were based on visual observations such as a lack of decoloration in transgenic leaves under stress conditions [13], [14] and chlorophyll content in salt stressed leaves [16]. In this work, we reported redox effects of Ced-9-expression in soybean hairy roots under stress conditions (Figure 5 and 6). Increases in antioxidant capacity in K599-empty hairy roots (Figure 5C) could indicate a response to oxidative stress induced by hairy root death-inducing conditions (Figure 5A and 5B); while no changes were observed between treatments in K599-CED9 hairy roots (Figure 5A, 5B, 5C and 5D). These results demonstrated that the expression of Ced9 prevents ROS generation in hairy roots under stress conditions.

On the other hand, the mammalian homologous of CED-9 may regulate metabolic efficiency in neurons through interaction with the mitochondrial F_1_F_0_ ATP synthase in the inner membrane [54]. Likewise, Qiao et al [13] suggested a possible contribution of Bcl-xL and Ced-9 to improved mitochondrial membrane potential when were expressed in plants. In this regard, this work demonstrated that K599-CED-9 hairy roots had improved metabolism assessed as ATP content (Figure 6), particularly in severe salt conditions.

Strikingly, despite of improved metabolism and tolerance to death-induced stress conditions, K599-CED9 hairy roots had a significant inhibition of its nodulation capacity (Figure 7). Moreover, given that cell death process is an early control of the number of nodules [55], [56], we expected that the expression of Ced-9 could impact positively on the nodulation process. Taking into account that one of the main action of Ced-9 is the ionic flux control, it is possible that its expression in legume could adversely affect the ion flux signatures that occur during rhizobium perception [57], [58]. Likewise, It has been reported in animals that CED9 interact with proteins involved in vesicular traffic and autophagy [59], which in turn have participation in organogenesis events [60]–[62]. In this regard, we have the hypothesis and also relevant unpublished data showing that CED9 expression, which have no homologues identified in plants, could affect nodule organogenesis by interacting with vesicular traffic and autophagy proteins conserved in plants.

In summary, in this work we characterized the effects of Ced-9-expression on soybean hairy root under different, ordered-like and necrosis-like root hair and root death-inducing conditions. In this respect, we demonstrated that part of improved tolerance given by Ced-9 expression is based on the maintenance of ionic and redox homeostasis capacity. However, contrary to expectations, Ced-9 expression drastically inhibited nodule formation, and consequently the expression of animal cell death suppressors seems not to be an adequate strategy to increase the nitrogen content derived from biological fixation.

## Materials and Methods

### Bacterial strain and plant material

Soybean seeds (*Glycine max* L. DM4800) were disinfected with sodium hypochlorite 5% (v/v) for 5 min and germinated in the dark for 48 h on filter paper moistened with distilled water. The seeds were incubated at 28 and 37 °C during the first and second 24 h periods, respectively, to promote the growth of roots and root hairs. *Bradyrhizobium japonicum* USDA 138 was cultured in yeast extract mannitol (YEM) medium [63] at 28 °C with constant agitation for 5 days (3×10^9^ cells mL^−1^). The bacteria were washed and resuspended in sterile water.

### Binary vector and *A. rhizogenes* strains

The binary vector pBI2113-Ced-9 has an efficient promoter cassette overexpressing the Ced-9 gene (GenBank accession number L26545), a *Caenorhabditis elegans* homolog of Bcl-xL which was kindly provided by Dr. Yuko Ohashi [12], [64]. Cucumopine-type *A. rhizogenes* strain K599 was used to infect cotyledon axes regions. *A. rhizogenes* K599 with pBI2113-Ced-9 was grown in Luria-Bertani (LB) medium containing kanamycin (Km) at 50 µg mL^−1^. To get fresh cells, *A. rhizogenes* K599 was grown on LB plates containing Km and incubated 48 h at 28 °C. Cells were collected from these plates and diluted into 1 mL of sterile water. For control hairy roots (K599-empty), a fresh culture of *A. rhizogenes* K599 lacking the binary vector was grown in LB medium without antibiotics.

### *A. rhizogenes*-mediated root transformation

Induction of *A. rhizogenes*-mediated root transformation protocol was modified from Estrada-Navarrete [27]. Briefly, after germination, sprouts were inoculated by injection directly into the cotyledonary nodes with a syringe and transferred to a hydroponic double tube system and incubated in a growth chamber under 16 h photoperiod (350 µmol m^−2^ s^−1^) at 26±2 °C. The smaller tube contained the sprout watered with B&D solution [65] supplemented with 8 mM KNO_3_ and it was within a larger tube that serves as moist chamber. Typically, soybean plants infected by *A. rhizogenes* started to show tumors approximately 5 days after inoculation. Twelve days after *A. rhizogenes* infection, plantlets exhibited numerous induced hairy roots per wound site. Primary root was removed from the plant by cutting approximately 1 cm below the cotyledon nodes and the composite plants were placed in plastic trays with B&D solution with or without KNO_3_ depending on the treatment to be performed.

### Root hairs death-inducing conditions

After germination, sprouts were incubated 30 min in aerated tubes that contained sterile water (control), *B. japonicum* (inoculated), 50 mM NaCl, *B. japonicum* in presence of 50 mM NaCl and 150 mM NaCl. Root hairs from roots subjected to different stress treatments were extracted by peeling the root zone containing young root hairs, which were immediately frozen in liquid air. Peeling was performed under a magnifying glass by making an incision with a scalpel in root and pulling the epidermal tissue containing the root hairs using a fine-tipped clamp. Root hairs of approximately 200 roots generate sufficient material for a sample.

### Hairy roots death-inducing conditions

Once primary root was removed from the plant by cutting below the cotyledon nodes, the composite plants were placed in aerated plastic trays with B&D solution supplemented with 8 mM KNO_3_ and incubated in a growth chamber under 16 h photoperiod (350 µmol m^−2^ s^−1^) at 26±2 °C during two weeks. Hairy roots were subjected to stress treatments for 3 h and then, they were immediately frozen in liquid air.

### Cell death evaluations

Cell death evaluations were performed by Evans Blue staining and DNA degradation analysis. Evans Blue is a dye used in the determination of cell viability [13] due to its inability to permeate intact cell membranes. When cells lose the membrane potential, the dye diffuses within the cell and it may visualized by conventional microscopy. The roots were incubated 10 min with Evans Blue 0.05% (w/v) in water or each NaCl levels assayed.

Genomic DNA was isolated using CTAB [66]. In brief, the samples were homogenized to a fine powder using a mortar and pestle under liquid nitrogen and thawed in CTAB extraction buffer (2% w/v CTAB, 1.4 M NaCl, 20 mM EDTA, 100 mM TRIS-HCl pH 8.0). RNase A was added and the homogenate was incubated for 30 min at 37 °C. DNA was extracted twice with an equal volume of chloroform:isoamylalcohol (24∶1 v/v) and precipitated with 0.6 vols. of isopropanol. For visualization of the DNA degradation, equal amounts of DNA (2 µg) were loaded on a 2% TAE agarose gel and stained with ethidium bromide.

The nuclear morphology of hairy roots was evaluated by acridine orange (AO, 50 µg/mL) and ethidium bromide (EtBr, 50 µg/mL) staining and observed with Zeiss confocal microscopy. AO and EtBr are dyes that intercalate DNA and fluoresce under UV light. The orange color and the presence of the dispersed chromatin in the cytoplasm indicate that the cells have lost integrity of the nuclear membrane and are in a very late stage of death. Both dyes are excited by 488 nm and emission was observed at 500–530 nm and 565–615 nm for AO and EtBr, respectively.

### MDA and ATP quantification in root hairs and hairy roots

The samples were homogenized using a mortar and pestle under liquid nitrogen and thawed in 3% (v/v) trichloroacetic acid (TCA) then centrifugation was carried out at 13,000 g, 4 °C during 15 min.

MDA levels were quantified according Heath and Packer [67]. Briefly, 100 µL of sample were mixed with 100 µL of 20% TCA +0.5% thiobarbituric acid (TBA), incubated at 90°C for 20 min and ice cold rapidly. The mix was centrifuged at 13,000 g for 10 min. The absorbance of the supernatant was read at 532 nm y 600 nm.

The concentration of ATP present in each of the samples was determined with a GloMax luminometer using a bioluminescent detection reagent (ENLITEN rLuciferase/Luciferin; Promega) according to manufacturer. The amount of ATP present in the sample was calculated from the measured relative light units using a standard curve spanning the relative light unit range obtained from the samples [68].

### Na^+^, K^+^ and Ca^+^ determination in hairy roots

Ion quantification was performed by high pressure liquid chromatography (Shimadzu LC2010) with Shim-pack IC–C3 column and non-suppressed system. Hairy roots segments (50 mg) were placed into 1 mL of 0.1 N nitric acid during 3 days. Samples were passed through 0.22 µm pore size MF Millipore cellulose membrane filters and diluted 6 times with MQ water. Then, 30 µL of samples were analyzed. The mobile phase was oxalic acid 2.5 mM and the time of chromatography was 20 min with a flow rate of 1.2 mL min^−1^. Quantitative analysis was done with multicationic standards by software LCSolution.

### Fluorometric H_2_O_2_ determination in hairy roots

The samples were homogenized in 3% (v/v) trichloroacetic acid (TCA) then centrifugation was carried out at 12,000 g, 4 °C during 15 min. 100 µL of supernatant was mixed with determination buffer (100 mM potassium phosphate pH 7.4, 4.5 U/mL horse radish peroxidase (HRP) and 1 mM p-hydroxyphenilacetic acid). Duplicates were incubated with catalase in order to diminish unspecific fluorescence. Hydrogen peroxide fluorescence was measured with spectrofluorometer Shimatzu at 371–414 nm excitation and emission respectively [69].

### FRAP (Ferric Reducing Ability of Plasma) assay

The samples were homogenized using a mortar and pestle under liquid nitrogen and ethanol 80% was added. Then, centrifugation was carried out at 12,000 g, 4 °C during 10 min. 100 µL of supernatant was mixed with reaction buffer (5 mL of Acetate buffer 0.3 M pH 3.6; 0.5 mL of TPTZ 10 mM (2,4, 6 Tris (2 pyridyl) s-triazine) diluted in 40 mM HCl and 0.5 mL of FeCl_3_ 200 mM) in a microplate on ice. Samples were removed from the ice and read at 600 nm after 20 min. A standard curve with TROLOX was done to calculate FRAP capacity in samples [70].

### Ascorbic acid determination

Samples were prepared for ascorbate analyses by homogenizing material in 1 mL of 3% trichloroacetic acid. The homogenate was centrifuged at 10 000×g at 4°C for 15 minutes and the supernatant was collected for analyses of ascorbate. Total ascorbate content was determined according to Gillespie and Ainsworth [71] with modifications [72]. The reaction mixture for total ascorbate contained a 50 µL aliquot of the supernatant, 15 µL of 150 mM phosphate buffer (pH 7.4) and 15 µL of 10 mM DTT; samples were incubated at room temperature for 10 min. After that, the mix was incubated for 60 min at 37 °C with 15 µL of 0.5% N-ethylmaleimide (NEM), 16.6% orthophosphoric acid (H_3_PO_4_), 1.33% α-α'bipyridyl, and 30 µL of 3% FeCl_3_. The samples were measured at 525 nm in ELISA MRX II. Reduced ascorbic acid content was determined using the same protocol, except for the addition of NEM and DTT.

### Protein content

Protein content was determined spectrophotometrically at 578 nm according to Bradford [73] with bovine serum albumin (BSA) as a standard.

### Evaluation of nodulation in hairy roots

Primary soybean root was removed twelve days post-infection with *A. rhizogenes* by cutting approximately 1 cm below the cotyledon nodes and the composite plants were placed in aerated plastic trays with B&D solution with 8 mM KNO_3_ in a growth chamber under 16 h photoperiod (350 µmol m^−2^ s^−1^) at 26±2 °C. After two days, the nutrient solution was replaced by B&D without KNO_3_ and the inoculation with *B. japonicum* USDA138 was performed. The nodule number was assessed 14 days post-inoculation.

### RNA extraction

Samples were homogenized in a cold mortar with TRIzol Reagent (1∶10 µg plant tissue:µL reagent), mixed for 1 min and incubated at room temperature for 5 min. Then, 0.2 mL chloroform per mL of TRIzol Reagent was added and incubated at room temperature for 3 min. After incubation, the samples were centrifuged at 12,000 g at 4°C for 15 min and the aqueous phases were transferred to clean tubes. RNA was precipitated by adding 1 vol. of isopropanol, incubated at room temperature for 10 min and centrifuged at 12,000 g at 4°C for 15 min. The precipitate was washed with 70% ethanol and the samples were centrifuged again at 12,000 g, 4°C for 15 min. The precipitate was dried and resuspended in DEPC water and its concentration was quantified using a NanoDrop 3300 spectrometer (Thermo Scientific). Purified RNA was treated with DNase I (Invitrogen) to remove genomic DNA, according to the manufacturer's instructions.

### qPCR

RNA DNA-free (1 to 2.5 µg) was used with oligo(dT) for first strand cDNA synthesis using the Moloney Murine Leukemia Virus Reverse Transcriptase (M-MLV RT, Promega) according to the manufacturer's instructions. For each primer pair, the presence of a unique product of the expected size was checked on ethidium bromide–stained agarose gels after PCR reactions. Absence of contaminant genomic DNA was confirmed in reactions with DNase treated RNA as template. The qPCR reaction was performed using iQ Universal SYBR Green Supermix Bio Rad.

Amplification of actin (forward primer 5′-AACGACCTTAATCTTCATGCTGC-3′ and reverse primer 5′- GGTAACATTGTGCTCAGTGGTGG-3′) and EF1α (forward primer 5′-GGTCATTGGTCATGTCGACTCTGG-3′ and reverse primer 5′-GCACCCAGGCATACTTGAATGACC-3′) was used to normalize the amount of template cDNA. The gene-specific primer pairs employed for the detection of transcripts of Ced-9 was: forward primer 5′-CTACGAACGAGCAGAAGCTGAA-3′ and reverse primer 5′- CAAGCTGAACATCATCCGCCCA-3′. qRT-PCR was performed in thermocycler iQ5 (BioRad) at 59°C with iQ SYBR Green Supermix (BioRad), according to the manufacturer's instruction.

### Statistical analyses

Data were analyzed using analysis of variance (ANOVA) followed by the DGC test model with InfoStat software [74].

## Supporting Information

Figure S1 **K599-CED9 composite plants developed both transgenic and wild type hairy roots.** Development of transgenic (K599-CED9) and non-transgenic (K599-empty) hairy roots in a soybean K599-CED9 composite plant. A) Identification of K599-CED9 hairy roots by Ced-9 PCR (+: positive control, numbers from 1 to 5: independent hairy roots of the same K599-CED9 composite plant). B) Ced-9-expression levels in K599-CED9 hairy roots measured by qRT-PCR and normalized to elongation factor 1α (EF1α).
(TIF)

Figure S2 **Hairy roots death-inducing stress conditions.** A) Evans blue staining of K599-empty and K599-CED9 hairy roots showing loss of membrane integrity after 3 h of treatment with 150 mM NaCl. B) DNA degradation in hairy roots incubated 3 h under control and stress conditions. 1: Control, 2: inoculated with *B. japonicum* in presence of 50 mM NaCl, 3: 150 mM NaCl. 2 µg of DNA were loaded on a 2% TAE agarose gel and stained with ethidium bromide.
(TIF)

Figure S3 **Nuclear morphology in root hairs.** K599-empty (A, C, E) and K599-CED9 (B, D, F) hairy roots were subjected 30 min under control (A and B), inoculated with *B. japonicum* (C and D), and 50 mM NaCl (E and F), and root hairs nuclear morphology was evaluated. Images were taken with a Zeiss confocal microscope. The excitation was performed simultaneously at 488 nm and emission filter BP 500–530 IR and BP 565–615 IR for AO and EtBr, respectively. AO: acridine orange channel, EtBr: ethidium bromide channel, AO/EtBr: image overlay.
(TIF)
