# Supplementary figures and images for: Expression of Animal Anti-Apoptotic Gene Ced-9 Enhances Tolerance during Glycine max L.–Bradyrhizobium japonicum Interaction under Saline Stress but Reduces Nodule Formation
Source: PLoS One. 2014 Jul 22;9(7):e101747. doi: 10.1371/journal.pone.0101747 (PMC4106779; doi:10.1371/journal.pone.0101747)

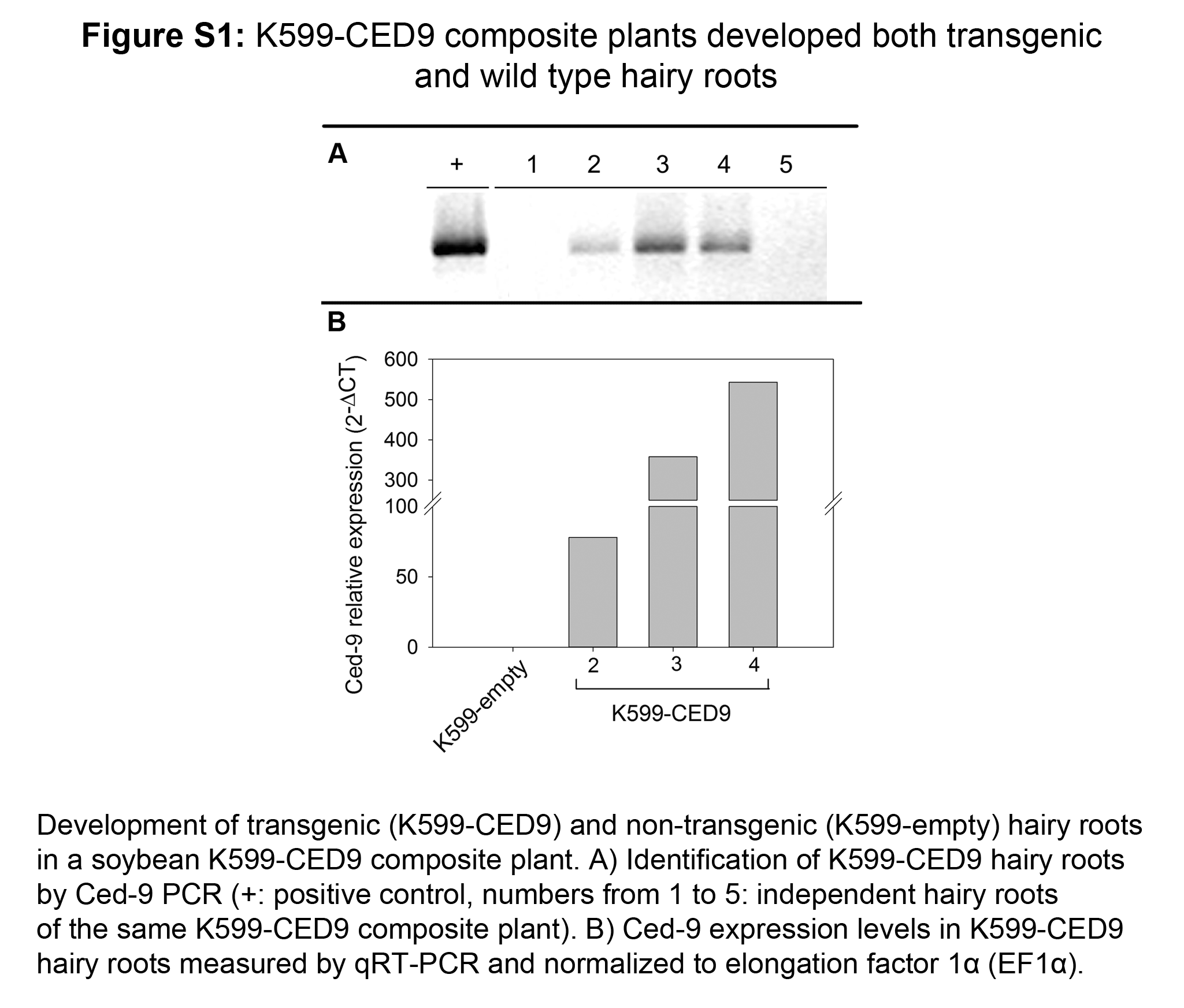

Supplement: Figure S1 — K599-CED9 composite plants developed both transgenic and wild type hairy roots. Development of transgenic (K599-CED9) and non-transgenic (K599-empty) hairy roots in a soybean K599-CED9 composite plant. A) Identification of K599-CED9 hairy roots by Ced-9 PCR (+: positive control, numbers from 1 to 5: independent hairy roots of the same K599-CED9 composite plant). B) Ced-9-expression levels in K599-CED9 hairy roots measured by qRT-PCR and normalized to elongation factor 1α (EF1α). (TIF) [file pone.0101747.s001.tif]

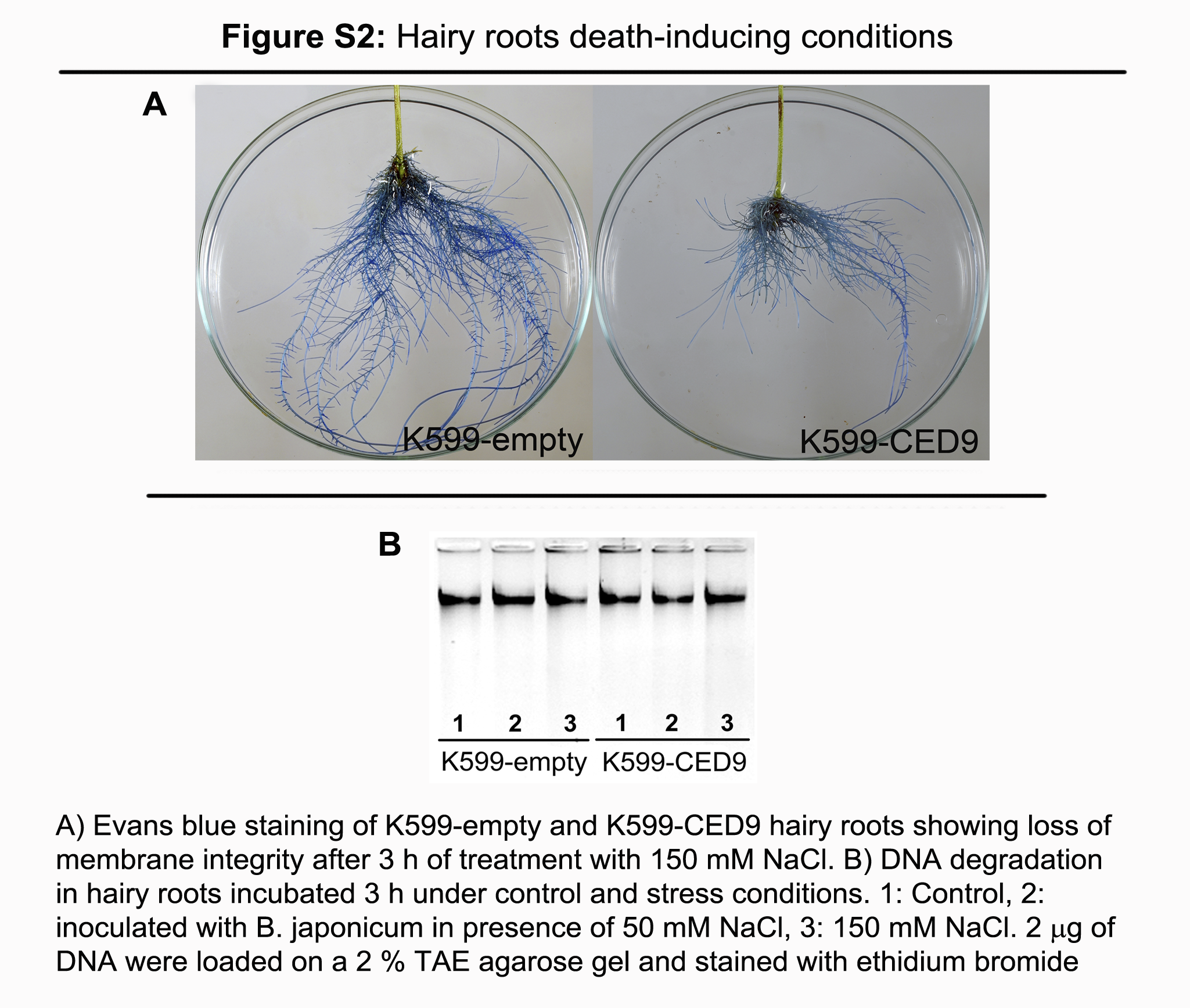

Supplement: Figure S2 — Hairy roots death-inducing stress conditions. A) Evans blue staining of K599-empty and K599-CED9 hairy roots showing loss of membrane integrity after 3 h of treatment with 150 mM NaCl. B) DNA degradation in hairy roots incubated 3 h under control and stress conditions. 1: Control, 2: inoculated with B. japonicum in presence of 50 mM NaCl, 3: 150 mM NaCl. 2 µg of DNA were loaded on a 2% TAE agarose gel and stained with ethidium bromide. (TIF) [file pone.0101747.s002.tif]

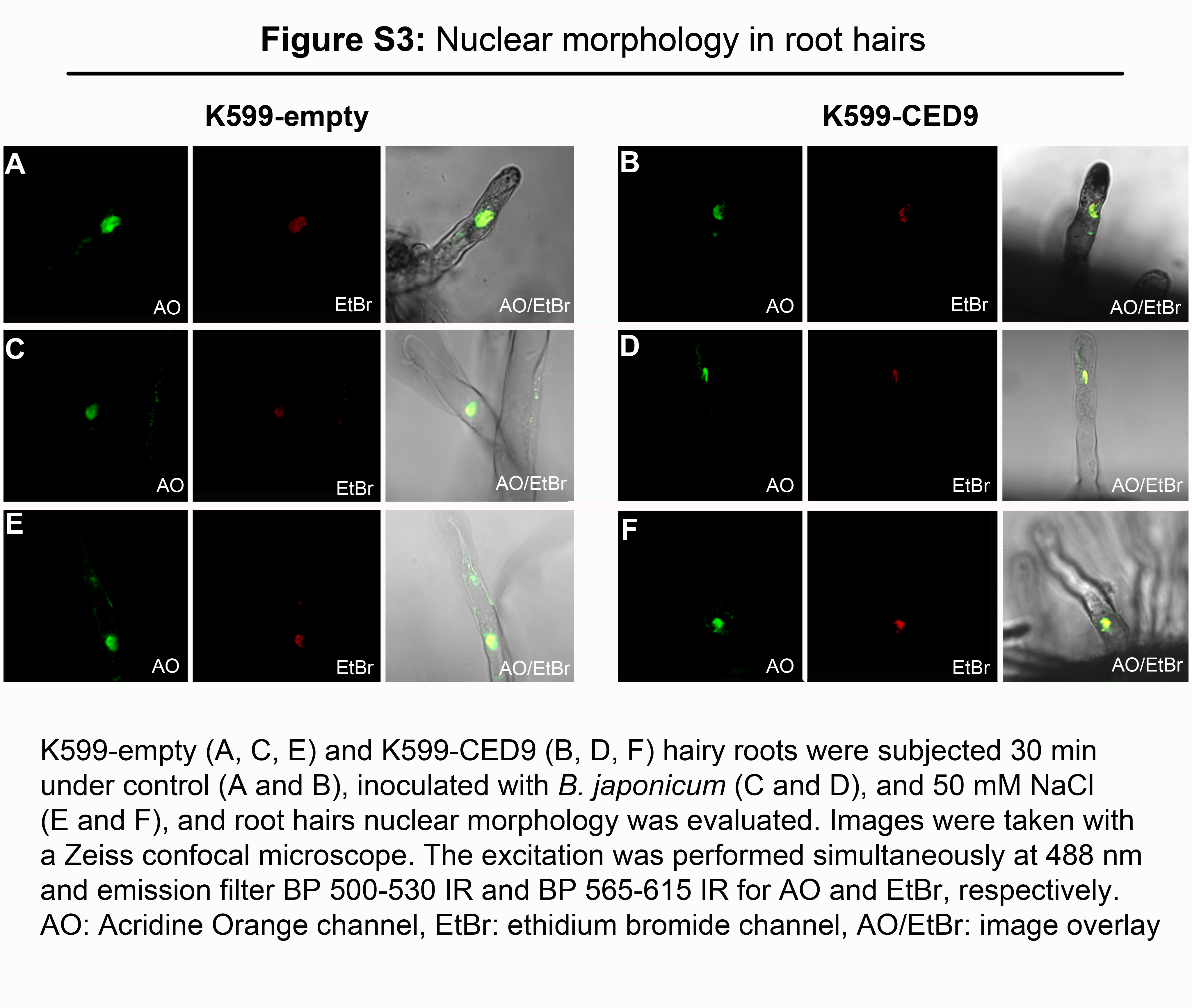

Supplement: Figure S3 — Nuclear morphology in root hairs. K599-empty (A, C, E) and K599-CED9 (B, D, F) hairy roots were subjected 30 min under control (A and B), inoculated with B. japonicum (C and D), and 50 mM NaCl (E and F), and root hairs nuclear morphology was evaluated. Images were taken with a Zeiss confocal microscope. The excitation was performed simultaneously at 488 nm and emission filter BP 500–530 IR and BP 565–615 IR for AO and EtBr, respectively. AO: acridine orange channel, EtBr: ethidium bromide channel, AO/EtBr: image overlay. (TIF) [file pone.0101747.s003.tif]
